# Comparative Analysis of the Bacterial and Fungal Communities in the Gut and the Crop of *Aedes albopictus* Mosquitoes: A Preliminary Study

**DOI:** 10.3390/pathogens9080628

**Published:** 2020-08-01

**Authors:** Morgane Guégan, Edwige Martin, Claire Valiente Moro

**Affiliations:** Univ Lyon, Université Claude Bernard Lyon 1, CNRS, INRAE, VetAgro Sup, UMR Ecologie Microbienne, F-69622 Villeurbanne, France; morganeguegan@free.fr (M.G.); edwige.martin@univ-lyon1.fr (E.M.)

**Keywords:** mosquito, microbiota, diverticula, crop, gut, *Aedes*

## Abstract

The Asian tiger mosquito *Aedes albopictus* is a major pathogen vector and one of the world’s most invasive species. In recent years, the study of mosquito-associated microbiota has received growing interest for reducing transmission of mosquito-borne pathogens. Most of studies on mosquito microbiota mainly focused on the gut bacteria. However, microorganisms can also colonize other organs and are not restricted to bacteria. In mosquitoes, the crop is the primary storage organ for sugars from the nectar feeding before it is transferred into the midgut for digestion. No study has yet investigated whether this organ can harbor microorganisms in *Ae. albopictus*. By using high-throughput sequencing, this study is the first to describe the microbiota including both bacteria and fungi in sugar-fed *Ae. albopictus* males and females. The results showed the presence of diverse and rich bacterial and fungal communities in the crop of both sexes that did not strongly differ from the community composition and structure found in the gut. Altogether, our results provide a thorough description of the crop-associated microbiota in *Ae. albopictus* which can open new avenues for further studies on trophic interactions between the mosquito and its microbiota.

## 1. Introduction

The Asian tiger mosquito *Aedes albopictus* (Skuse, 1894) is of growing public health concern worldwide [1]. Being able to transmit at least 22 arboviruses including dengue, chikungunya and Zika viruses [2], this species is also considered to be a major invasive species [3]. Native to southeast Asia, this mosquito has rapidly spread to all continents except Antarctica, including tropical as well as temperate areas [1]. Its invasion success has been favored by global trade and its singular ecological plasticity, allowing it to colonize a wide range of breeding sites ranging from natural habitats (cut bamboo or tree-holes) to artificial man-made containers (tires, buckets, etc.). In addition, some *Ae. albopictus* populations in temperate regions are able to lay overwintering eggs that are resistant to cold and desiccation, making this species highly adaptable [4].

In the last decade, there was an increasing interest in studying interactions between *Ae. albopictus* and its microbiota. Early studies were mainly descriptive and focused on bacterial communities and their variation factors [5]. In recent years, functional studies have emerged, demonstrating a role of bacteria in important traits of mosquito biology such as development, reproduction and vector competence [6,7,8]. Interestingly, an unsuspected diversity of fungal communities was recently highlighted in natural populations of *Ae. albopictus*, raising questions about their role in the mosquito [9]. Most studies on mosquito microbiota focused on the gut, as this organ is known to play a role in mosquito metabolism and immunity and is the first point of entry for transmitted viruses [10,11]. However, microorganisms can colonize other organs in mosquitoes, including reproductive tissues and salivary glands [12].

The composition and diversity in the ventral diverticulum or crop have been largely ignored, while this organ is important for mosquito nutrition. Sugar feeding is a fundamental characteristic of mosquito life. Both sexes feed on floral and extrafloral nectar as well as honeydew, which are the main sources of sugar, an important energy source for mosquitoes [13]. In particular, male mosquitoes require sugar sources for survival and reproduction [14]. After ingestion, the nectar is first stored in the crop as food reserves [10]. Then, sugars are slowly discharged from the crop to the midgut where the digestion occurs [15]. Salivary enzymes, ingested with saliva during the sugar meal, favor the solubilization of sugars and intra-cellular metabolism in the crop [16,17] but not the sugar digestion in the gut [15].

To our knowledge, there is very little information on the colonization of the crop by microorganisms. The high concentration of carbohydrates and the acidic pH in this organ could favor the development of specific microorganisms [5]. Using culture-dependent methods, Gusmão et al. [18] were the first to identify bacteria, including *Serratia* and *Bacillus*, as well as the yeast *Pichia* sp. in the crop of *Ae. aegypti*. They also demonstrated that these microorganisms could be transferred to the midgut along with food [19]. As far as we know, there is no study on the microbial colonization of the crop in *Ae. albopictus*. For this purpose, we conducted the first study to investigate if sugar-fed *Ae. albopictus* (both males and females) harbor bacteria and fungi in the crop by using culture-independent approaches and whether this microbial community differs from that found in the gut.

## 2. Results and Discussion

### 2.1. Microbial Diversity in the Gut and the Crop

The number of bacterial operational taxonomic units (OTUs) varied from 78 ± 7 (males) to 104 ± 39 (females) in the gut and from 73 ± 22 (females) to 80 ± 11 (males) in the crop. The number of fungal OTUs varied from 25 ± 3 (females) to 30 ± 7 (males) in the gut and from 24 ± 8 (males) to 24 ± 12 (females) in the crop. Multiple comparison analysis of the α-diversity was performed using the Shannon index (Table 1).

**Figure 1:**
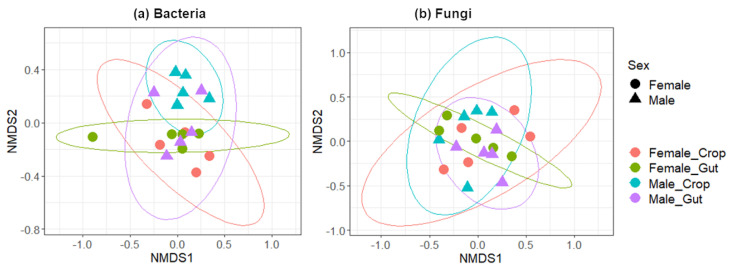
Non-metric multidimensional scaling (NMDS) plots on (**a**) bacterial and (**b**) fungal operational taxonomic units (OTUs) in relation to the sex and organ of mosquitoes. Females and males are represented with circles and triangles, respectively. Female crops, female guts, male crops and male guts are represented in red, green, blue and purple, respectively. Ellipses represent 95% confidence intervals of centroids for each point.

No significant difference was found in the α-diversity of microbial communities between guts and crops (ANOVA, *F* = 0.056, *p* = 0.816 for bacteria and *F* = 1.624, *p* = 0.221 for fungi) or between females and males (ANOVA, *F* = 0.818, *p* = 0.379 for bacteria and *F* = 1.051, *p* = 0.321 for fungi). The measures of the β-diversity by calculating the Bray–Curtis distances showed that neither the sex (Adonis-ANOVA, *R*^2^ = 0.05597, *p* = 0.238 for bacteria and *R*^2^ = 0.05614, *p* = 0.282 for fungi), nor the organ (Adonis-ANOVA, *R*^2^ = 0.04835, *p* = 0.598 for bacteria and *R*^2^ = 0.04582, *p* = 0.559 for fungi) affected bacterial and fungal community composition and structure (Figure 1). In mosquitoes, microbiota composition differences have been largely documented across different factors such as the mosquito species, the sex, the organ or the stage [5]. In this study, the lack of differences according to the sex of mosquitoes could be explained by the fact that mosquitoes were reared under controlled laboratory conditions and thus are exposed to much less diverse nutritional resources than those encountered by both sexes in the field. Thus, these results confirm that feeding habits play important roles in the composition and structure of their associated microbiota. However, bacterial communities were much more structured by sex than the fungal communities suggesting that gender-related factors differently influence the structure of bacteria compared to fungi. As fungal spores are an important component in ambient air, further studies are needed to evaluate which fungal species are truly commensal. Such knowledge could help to better characterize which factors shape the mycobiota community structure.

### 2.2. Taxonomic Composition of Bacterial and Fungal Communities in the Gut and the Crop

**Figure 2:**
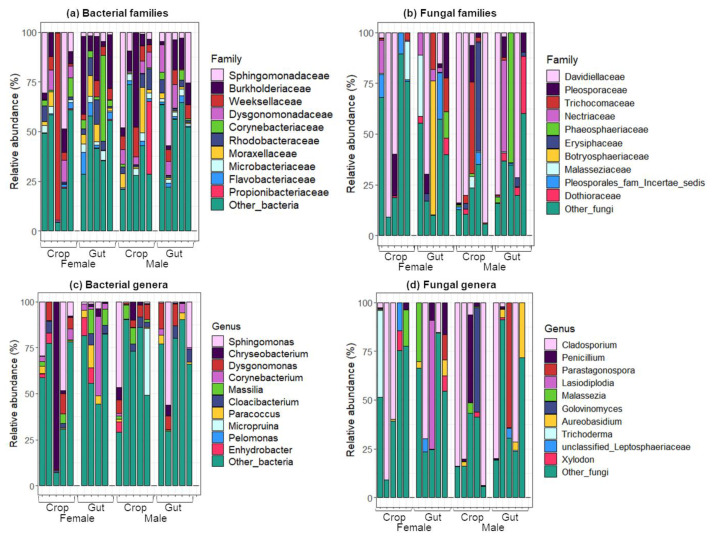
Relative abundance of the 10 most abundant taxa at the family or genus levels in the guts and the crops of mosquitoes of both sexes. Bacteria are shown in the panels (**a**) and (**c**), while fungi are shown in the panels (**b**) and (**d**).

Sequencing the 16S rRNA gene and fungal internal transcribed spacer (ITS) region was used for taxonomic identification of bacteria and fungi, respectively. At the phylum level, whatever the organ or the sex, Proteobacteria dominated bacterial communities followed by Bacteroidetes, Actinobacteria and Firmicutes. Regarding fungi, Ascomycota was the most prevalent phylum, followed by Basidiomycota. In females, Weeksellaceae and Burkholderiaceae were the most abundant families in crops (21.2%) and guts (14.3%), respectively (Figure 2a). Conversely, in males the Burkholderiaceae family was predominant in crops (19.8%), while Sphingomonadaceae (18.8%) dominated the guts. The Weeksellaceae family was more abundant in females than in males (21.2% and 4.2% in crops, Mann–Whitney–Wilcoxon, *W* = 12.5, *p* = 0.54; 6.5% and 4.2% in guts, *W* = 15, *p* = 0.3), and the Corynebacteriaceae family was more abundant in the crops than in the guts (2.5% and 0.6% in females, Mann–Whitney–Wilcoxon, *W* = 14, *p* = 0.42; 11% and 1.97% in males, *W* = 18, *p* = 0.15). The abundance of Dysgonomonadaceae was lower than 1% in the male crops, and the Propionibacteriaceae was found in relatively high abundance only in the female guts (7.3%). Even though the Davidiellaceae family dominated fungal communities irrespective of the sample considered, variations were highlighted in the abundance of other families according to the sex and organs (Figure 2b). For example, Trichocomaceae were more abundant in female guts than in crops (10.3% and 0.9%, Mann–Whitney–Wilcoxon, *W* = 5, *p* = 0.071) and in male crops than in guts (6.9% and 0.33%, Mann–Whitney–Wilcoxon, *W* = 13, *p* = 0.5). In males, the most abundant families were Phaeosphaeriaceae (2.6% and 13.4%) and Dothioraceae (2.3% and 7.4%) in the crops and guts, respectively. At the genus level, *Sphingomonas* dominated the bacterial communities (17.1%, 9.7% and 16.6%, in female crops, female guts and male guts, respectively) except for male crops where it was *Corynebacterium* (11%) (Figure 2c). These two bacterial genera have already been reported in some mosquito species, including *Ae. albopictus* [20,21,22,23]. *Sphingomonas* is widely distributed in the environment thanks to its ability to metabolize a wide variety of carbon sources and to survive with few nutrients [24]. In females, the most abundant genus was *Chryseobacterium* (18.8% and 3.8%, in the crops and guts, respectively) whereas *Corynebacterium* (11% and 1.9%, in the crops and guts, respectively) and *Paracoccus* (4.4% and 2%, in the crops and guts, respectively) were the most abundant genera in males. *Corynebacterium* was more abundant in the crops than in the guts (1.77% and 0.5% in females, Mann–Whitney–Wilcoxon, *W* = 10.5, *p* = 0.38; 11% and 1.9% in males, *W* = 18, *p* = 0.15) and *Chryseobacterium* was more abundant in females than in males (18.8% and 1.3% in crops, Mann–Whitney–Wilcoxon, *W* = 13.5, *p* = 0.46; 3.8% and 1.4% in guts, *W* = 20, *p* = 0.075). Interestingly, *Chryseobacterium* has been shown to be frequently associated with mosquito microbiota and is known to play important roles in mosquito development and microbial competition [25,26,27]. Concerning fungi, the genus *Cladosporium* dominated both organs for each sex (Figure 2d). This result is consistent with previous studies [9,28] where this fungus was both the most prevalent and abundant species of the whole mosquito mycobiota. The genera *Parastagonospora* (2.6% and 0% in crops, Mann–Whitney–Wilcoxon, *W* = 10, *p* = 0.212, 12.9% and 0.006% in guts, *W* = 12, *p* = 0.5) and *Aureobasidium* (2.3% and 0.2% in crops, Mann–Whitney–Wilcoxon, *W* = 9, *p* = 0.22; 7.4% and 0.48% in guts, *W* = 4.5, *p* = 0.053) were more abundant in males than in females. At the organ level, *Malassezia* (3.9% and 1.3% in females, Mann–Whitney–Wilcoxon, *W* = 10.5, *p* = 0.373; 6.1% and 0.11% in males, *W* = 15, *p* = 0.333) and *Xylodon* (1.9% and 0.5% in females, Mann–Whitney–Wilcoxon, *W* = 13, *p* = 0.5; 1.5% and 0.3% in males, *W* = 9, *p* = 0.24) were more abundant in the crops than in the guts. Interestingly, the species *Aureobasidium pullulans* was reported in the top five fungal species found in natural populations of *Ae. albopictus* from different geographic origins [9]. More generally, when describing microbial community composition through DNA, it is not possible to conclude with certainty whether the microorganisms detected are alive. Isolating microorganisms from these two organs could provide insights into their metabolic and physiological properties as well as their potential contribution for the mosquito host. Interestingly, recent studies highlighted that live bacteria and fungi, which are associated with mosquitoes, are able to stimulate larval growth only when viable and present above a certain density [29,30]. It was also shown that mosquitoes could survive in the absence of a living microbiota, suggesting that the gut living microbiota could favor mosquito physiological properties by participating in their nutrition [31]. Moreover, it is important to note that experiments were performed on lab-reared mosquitoes in a controlled laboratory environment. Additional experimental data are needed to deeply investigate how similar or different the gut and crop microbiota composition is in field mosquito populations, which are exposed to a variety of sugar sources, and how it impacts physiological processes of the mosquito. Indeed, as previously reported, larval or adult diet is an important factor that shapes the microbiota composition [32,33].

### 2.3. Shared Microbiota between the Gut and the Crop

**Figure 3:**
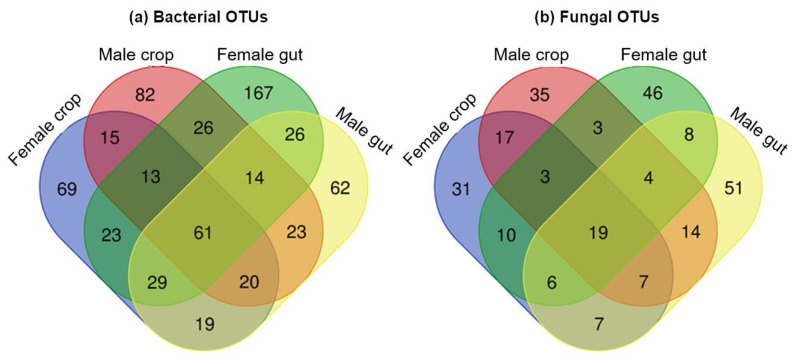
Venn diagram showing specific and common (**a**) bacterial and (**b**) fungal operational taxonomic units (OTUs) between crops and guts in female and male *Ae. albopictus*.

A total of 61 bacterial and 19 fungal OTUs (i.e., 9.4% and 7.3%, respectively, of the total OTUs) was shared by both organs in females and males (OTUs found at least in one individual mosquito organ of each sex) (Figure 3a,b). Among these shared OTUs, 28, 15, 9 and 9 belonged to the bacterial phyla Proteobacteria, Actinobacteria, Bacteroidetes and Firmicutes, respectively, and 14 and 4 OTUs belonged to the fungal phyla Ascomycota and Basidiomycota, respectively. Concerning bacteria, 166 and 255 OTUs (i.e., 25.6% and 39.3%) were specific to the crop (31.9% Proteobacteria, 19.3% Bacteroidetes and 28.9% Actinobacteria) and the gut (54.9% Proteobacteria, 15.3% Bacteroidetes and 12.1% Actinobacteria), respectively, and 259 and 167 OTUs (i.e., 39.9% and 25.7%, respectively) were specific to females (49% Proteobacteria, 16.2% Bacteroidetes and 18.1% Actinobacteria) and males (41.3% Proteobacteria, 16.8% Bacteroidetes and 20.9% Actinobacteria), respectively. Concerning fungi, 83 and 105 OTUs (i.e., 31.8% and 40.2%, respectively) were specific to the crop (44.6% Ascomycota and 38.5% Basidiomycota) and the gut (50.5% Ascomycota and 41.9% Basidiomycota), respectively, and 87 and 100 OTUs (i.e., 33.3% and 38.3%, respectively) were specific to females (52.9% Ascomycota and 37.9% Basidiomycota) and males (43% Ascomycota and 48% Basidiomycota), respectively. A list of common or specific OTUs according to the sex or the organ is given in Table S1. Previous studies reported shared bacteria between different mosquito organs such as the midgut, the reproductive organs and salivary glands [34,35]. Interestingly, we showed the presence of conserved group of bacteria between the crop and the gut of *Ae. albopictus* mosquito individuals. This observation is consistent with the previous study of Gusmão et al. [18], where they showed the transfer of microorganisms from the crop to the midgut in *Ae. aegypti* mosquitoes. So far understudied, our study shows that fungi also colonize different mosquito tissues. This suggests that some microorganisms, including both bacteria and fungi, exhibit wide colonization ability as previously observed [35]. However, some bacterial and fungal genera were also found to be specific to one or another tissue, reflecting potential organ-microorganism adaptations. This could be explained by specific local physicochemical conditions encountered in each tissue at the scale of the individual. For instance, the mosquito midgut has a pH regulated to pH 6 [36], while the pH in the crop dissected right after sugar feeding is close to 6.5 [18]. Moreover, contrary to the gut, the crop is not directly exposed to the blood flux. Given some taxon specificities, further studies are needed to evaluate whether bacteria and fungi could be involved in the metabolization of sugars present in the crop, providing important nutrients to adult physiology. In addition, it was previously demonstrated that (i) after female mosquitoes ingested a blood meal, a small amount of blood could also be partially diverted to the crop [37], (ii) the more sucrose in the blood, the greater the amount discharged into the crop [38] and (iii) arboviruses infection may occur in the crop of mosquito vectors [38]. Altogether, these observations stress the importance of studying multipartite interactions between the pathogen, the mosquito and its microbiota in this organ. Knowledge on this specific topic could have direct implications in the development of new vector control methods.

## 3. Materials and Methods

### 3.1. Mosquito Colony and Rearing

All experiments were performed on the generation F5 of a laboratory mosquito colony originally from the French island of Réunion. Mosquito larvae were reared in dechlorinated water at 25 °C under a 16:8 h L:D photoperiod. They were fed daily a mixture of 75/25% blend of fish food (TetraMin^®^, Melle, Germany) and yeast tablet (Biover^®^, Nazareth, Belgium). Adults were fed with 10% sucrose and reared at 28 °C, 80% relative humidity, under a 16:8 h L:D photoperiod. At 10 d post-emergence, 5 males and 5 females were isolated and starved for 12 h. The next day, they received 10% fructose and after sugar feeding, individuals were killed in a freezer and used for dissections.

### 3.2. DNA Extraction

Prior to dissection for crop and gut recovery, mosquitoes were surface-sterilized as previously described [39]. For each individual, the crop and the gut were separated from the rest of the body under aseptic conditions and individually placed in tubes containing sterile 1X phosphate buffered saline solution (PBS, Life Technologies, NY, USA). Genomic DNA was extracted from each organ individually using the DNeasy Blood and Tissue kit (Qiagen, Hilden, Germany), as previously described [40] and stored at −20 °C. The DNA was quantified using the UVmc2 spectrophotometer (SAFAS, Monaco).

### 3.3. DNA Library Preparation, MiSeq Illumina Sequencing and Data Analysis

For the identification of bacterial and fungal communities, PCR amplifications were performed in triplicate, purified and quantified as previously described [41]. Biofidal (Vaulx-en-Velin, France) performed the library construction and the next-generation sequencing (2 × 300 bp paired-end Illumina MiSeq run). All fastQ files are available under the project accession number PRJEB39124 at EMBL European Nucleotide Archive (https://www.ebi.ac.uk/ena). A total of 1,427,727 and 1,214,901 reads were obtained and demultiplexed for bacteria and fungi, respectively. The quality control and sequence analyses were performed using the FROGS pipeline [42], as previously described [27]. The taxonomic affiliation of OTUs was performed using the Mothur pipeline [43] by clustering sequences at a level of 97% similarity according to the median neighbor method at 80% minimum bootstrap using a naïve Bayesian classifier [44] using the SILVA 132 [45] and the ITS UNITE [46] databases for bacteria and fungi, respectively. Contaminants were filtered out using the negative controls (blank extraction and PCR), as previously described [41]. Normalization was performed at 5254 and 3140 sequences for the bacterial and fungal sequences, respectively, and a total of 649 bacterial OTUs and 261 fungal OTUs were obtained. Data analysis, including α and β diversity and statistical tests (Mann–Whitney–Wilcoxon and Adonis-ANOVA), were performed with R software [47] using the packages phyloseq [48], vegan [49] ggplot2 [50], ape [51] dplyr [52], ggrepel [53] and plyr [54]. Venn diagrams were carried out with the Venn Diagrams software from the Van de Peer Lab Bioinformatics and Evolutionary Genomics (http://bioinformatics.psb.ugent.be/webtools/Venn/).

## 4. Conclusions

This exploratory study describes for the first time the composition of microbial communities harbored in the crop of *Ae. albopictus* mosquitoes. The identification of a shared microbiota between the crop and the gut, two important organs in mosquito nutrition, prompt further studies to gain insight into trophic interactions between mosquito and its microbiota. Interestingly, a recent study identified bacteria and fungi assimilating fructose within the gut of this mosquito species [41]. Further studies should be considered in the future taking into account the sugar trajectory in both the crop and the gut as well as the involvement of microorganisms in sugar metabolism.

## Figures and Tables

**Table 1:** α-Diversity of bacterial and fungal communities represented by the Shannon index.

Microorganism	Mosquito Sex	Organ	Shannon Index
Bacteria	Female	Crop	2.74 ± 1.21
Bacteria	Female	Gut	3.17 ± 0.59
Bacteria	Male	Crop	3.42 ± 0.39
Bacteria	Male	Gut	3.17 ± 0.56
Fungi	Female	Crop	1.73 ± 0.82
Fungi	Female	Gut	1.22 ± 0.56
Fungi	Male	Crop	1.97 ± 0.64
Fungi	Male	Gut	1.65 ± 0.53

## Supplementary Materials

The following are available online at https://www.mdpi.com/2076-0817/9/8/628/s1, Table S1: List of bacterial and fungal OTUs found in common or specific to the sex or the organ.
